# A distributed software system for integrating data-intensive imaging methods in a hard X-ray nanoprobe beamline at the SSRF

**DOI:** 10.1107/S1600577524006994

**Published:** 2024-08-22

**Authors:** Peicheng Zhang, Zhisen Jiang, Yan He, Aiguo Li

**Affiliations:** a Shanghai Advanced Research Institute, Chinese Academy of Sciences Shanghai201210 People’s Republic of China; b School of Physical Science and Technology ShanghaiTech University Shanghai201210 People’s Republic of China; ESRF – The European Synchrotron, France

**Keywords:** distributed software system, synchrotron radiation big data, ptychography, parallel computing

## Abstract

The development of hard X-ray nanoprobe techniques has given rise to a number of experimental methods, like nano-XAS, nano-XRD, nano-XRF, ptychography and tomography. Each method has its own unique data processing algorithms. With the increase in data acquisition rate, the large amount of generated data is now a big challenge to these algorithms. In this work, an intuitive, user-friendly software system is introduced to integrate and manage these algorithms; by taking advantage of the loosely coupled, component-based design approach of the system, the data processing speed of the imaging algorithm is enhanced through optimization of the parallelism efficiency. This study provides meaningful solutions to tackle complexity challenges faced in synchrotron data processing.

## Abstract 2

The development of hard X-ray nanoprobe techniques has given rise to a number of experimental methods, like nano-XAS, nano-XRD, nano-XRF, ptychography and tomography. Each method has its own unique data processing algorithms. With the increase in data acquisition rate, the large amount of generated data is now a big challenge to these algorithms. Here, software is proposed that is based on distributed systems with an intuitive graphical user interface design that allows users to easily operate and configure the software.

## 1. Introduction

The hard X-ray nanoprobe beamline at the Shanghai Synchrotron Radiation Facility (SSRF) offers a lot of high spatial resolution methods for structure, morphology and composition studies of heterogeneous systems. Coherence diffraction imaging (CDI) and ptychography are based on the coherence of light to achieve high spatial resolution, while scanning diffraction, X-ray fluorescence (XRF) and X-ray absorption near-edge structure spectroscopy (XANES) mapping are based on a focused beam (Johansson *et al.*, 2021 ▸; Quinn *et al.*, 2021 ▸; Martínez-Criado *et al.*, 2016 ▸; Leake *et al.*, 2019 ▸).

With the continuous advancements in light sources, detectors and automation capabilities at beamlines, the significant increase in data acquisition rates poses new challenges for beamline scientists and users in efficiently managing and analysing the growing volume of data (Blair *et al.*, 2014 ▸; Wang *et al.*, 2018 ▸).

The limitations on the efficiency of experiments are no longer primarily related to data acquisition, but rather to data processing (Deslippe *et al.*, 2014 ▸). This trend is particularly evident in experimental methods like tomography, ptychography and artificial intelligence algorithms based on deep learning, which are increasingly applied to synchrotron imaging technologies (Pfeiffer, 2018 ▸; Dierolf *et al.*, 2010 ▸). There is a noticeable shift towards data-intensive algorithms. Addressing the challenge of enhancing data storage and processing capabilities to accommodate the exponential growth in dataset size has become an urgent issue that merits further research (Bicer, 2014 ▸; Bicer *et al.*, 2017 ▸).

Over the past decade, GPU-based heterogeneous computing technology has become widely used for parallel computing tasks on large-scale general scientific data sets, owing to its exceptional parallelism and computing capabilities. Its application in tomography and ptychography has also been explored to a certain extent; however, most existing methods are optimized for individual processing units (Pelt *et al.*, 2016 ▸) or local multiple processing units (Favre-Nicolin *et al.*, 2020 ▸), without specific optimization for cluster-based multiple processing units. With the continuous improvement in the brightness of the light source beam, the disparity in speed between data acquisition and data processing components is becoming increasingly pronounced. These single-image processor systems are no longer sufficient to meet the processing requirements of future data-centric computational imaging applications.

In the field of synchrotron light sources, free-electron lasers and other large-scale imaging scientific facilities, the prevailing big data challenges encompass various aspects, including but not limited to data storage, data transmission and high-speed data analysis. Large scientific facilities such as the Advanced Light Source (ALS) (Pandolfi *et al.*, 2018 ▸; Dede *et al.*, 2013 ▸), the Advanced Photon Source (APS) (Gürsoy *et al.*, 2014 ▸), the Swiss Light Source (SLS) (Buurlage *et al.*, 2019 ▸) and the European Synchrotron Radiation Facility (ESRF) (Mirone *et al.*, 2014 ▸; Vogelgesang *et al.*, 2012 ▸, 2016 ▸) have all put foward their respective solutions to tackle the substantial big data challenges they currently face.

On the other hand, the nanoprobe beamline has a wide variety of characterization methods. Thus, for the convenience of users, it is important to create a platform with a user-friendly interface that integrates multiple algorithms.

Therefore, we propose a software based on distributed systems with an intuitive graphical user interface design that allows users to easily operate and configure the software. This software integrates multiple imaging methods into a user-friendly platform. Furthermore, we have adopted a distributed computing approach to decompose the imaging task into multiple subtasks and distribute them to multiple computing nodes for parallel processing. By using an event-driven programming model, image data can be updated and displayed. We have also implemented efficient data transmission protocols and compression methods to minimize data transmission latency, thereby enhancing the parallelism performance of imaging algorithms.

## 2. Method

**Figure 1:**
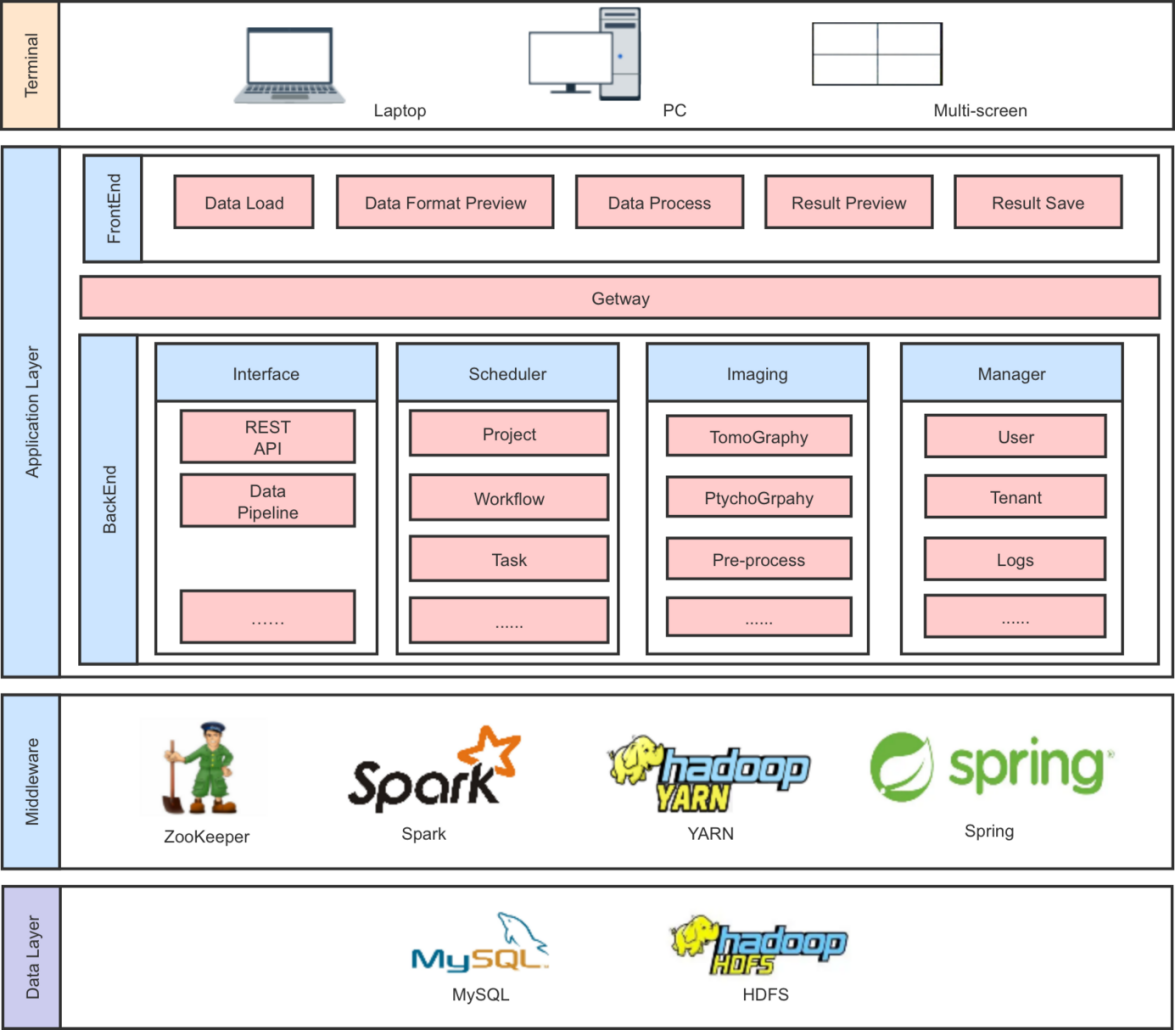
The architecture of the software system. Three main layers are included: application layer, middleware and data layer.

This section provides a detailed description of the software system architecture (shown in Fig. 1 ▸) designed to address the core objectives of user-friendliness and algorithm integration as well as the principles and methodologies involved in the implementation. The discussion includes two main aspects: the realization of algorithm integration and user-friendliness, and the implementation of algorithm parallelism optimization.

We applied some classical design principles from the fields of human–computer interaction (HCI) and user experience (UX) to enhance the user-friendliness of the software system (Norman, 2013 ▸; Krug, 2000 ▸).

Firstly, we simplified the user interface. Because of the complexity of the computational imaging tasks and the diversity of imaging algorithm parameters, existing imaging software tends to employ cumbersome and intricate user interfaces (Marone *et al.*, 2017 ▸). The advantage of such a design is its capability to offer users fine-grained control over customization of their imaging tasks. However, for users with limited exposure to computational imaging, this design introduces significant cognitive load and reduced clarity. Therefore, simplifying the user interface was our initial step. We designed an intuitive graphical user interface (GUI) implemented using *Vue.js* and *Node.js*. This GUI offers convenient access to various system functionalities, including:

*Data loading*: offering a simplified interface for seamless data importation.

*Data preview*: allowing users to preview raw data sets and processed data sets to effectively understand their characteristics, structure and outcomes.

*Data processing*: providing a rich set of data processing features, including operations such as filtering, denoising and image reconstruction. These operations are configurable and adjustable through the user-friendly interface.

**Figure 2:**
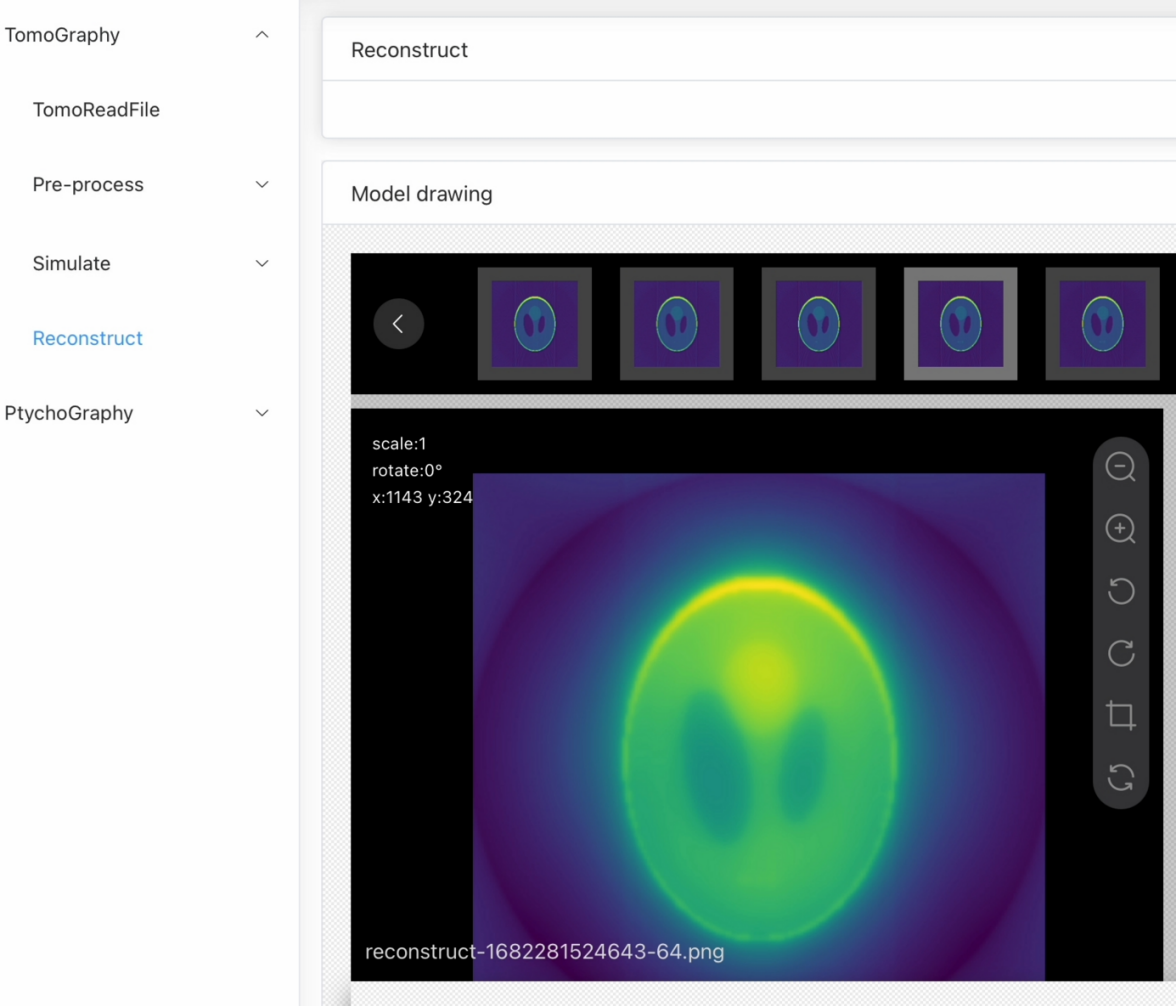
The user interface for algorithm selection, parameter selection and display of results.

As shown in Fig. 2 ▸, the user interface for performing tomographic reconstruction consists of four parts. The top panel offers users various system-wide functionalities, such as documentation and token authentication. The left panel serves as the function selection area, allowing users to switch between imaging methods and various data processing steps. The upper right panel is designated for parameter selection, enabling users to modify or select parameters for data processing. Finally, the model drawing panel provides a preview of the results obtained after data processing.

Subsequently, in order to reduce the user’s learning curve and minimize the errors that may be caused by manual operation, we form a set of generalized data processing procedures and parameter configurations based on the summaries of past experimental methods. Algorithm engineers can predefine the algorithms and workflows that users are likely to use and deploy them to the system. The process of cluster deployment and execution of algorithms is transparent to the user, who has no knowledge of the complex computational processes involved in the imaging task. The user simply selects the data set, enters the parameters, submits the task, and then waits for the server to complete the execution. For our algorithm engineers, maintaining algorithms and configuring and deploying workflows for specific imaging methods are the only necessary tasks. Task submission and execution, scheduling between multiple algorithms, system response to the user, and data read/write operations are all transparent to the algorithm engineers. In short, the user is responsible for dataset and parameter selection and results analysis; the algorithm engineer is responsible for algorithm and workflow maintenance; and all other tasks are handled by the system.

**Figure 3:**
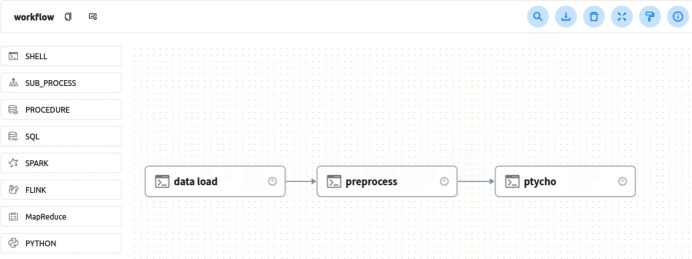
Visualization interface for customized data processing.

Of course, for those who want to take the data processing into their own hands, we also provide powerful algorithmic extension capabilities. This algorithm scalability is achieved through the abstraction and modularization of the algorithm operation process. Users can extend the functionality of the software by directly integrating their own custom processing algorithms through our modular architecture. This modular approach allows for the seamless integration of custom scripts or algorithms written in popular programming languages such as Python, Shell or similar environments. In terms of algorithm configuration, as shown in Fig. 3 ▸, we provide a visual interface that allows users to integrate their own algorithms into the system through simple operations and to easily customize the algorithm operations already in the system. This approach not only enhances the flexibility of our software, but also enables users to independently adapt and optimize their data analysis pipelines. We also hope to collaborate with experts and peers in maintaining and developing various parts of the system, especially the algorithms.

To achieve algorithm integration, two key modules were introduced: the Imaging module and the Scheduler module.

*Imaging module*: focused on implementing diverse imaging algorithms such as tomography and ptychography, supplemented by data preprocessing techniques to optimize imaging results.

*Scheduler module*: implemented using Apache *DolphinScheduler*, serving as a workflow scheduling framework. It facilitates effective management and scheduling of tasks, allowing the combination of tasks like shake alignment and reconstruction into flexible imaging workflows.

To improve the efficiency of the algorithm, we also optimized the parallelism computing method. Parallel computing involves using multiple processes simultaneously to perform an imaging task, resulting in a significant increase in computational efficiency. Most existing imaging software has already implemented optimizations for GPU computing. However, optimization for multiple GPUs, especially in a clustered environment, is still underdeveloped. The existing optimizations primarily focus on local multiple GPUs, leveraging advantages such as reduced resource consumption on network I/O and utilizing memory I/O for higher performance (Yu *et al.*, 2022 ▸). However, local multiple GPUs demand higher hardware performance and have lower fault tolerance (Dean & Ghemawat, 2008 ▸).

Our system adopts a cluster optimization approach based on the combination of the Spark engine and the YARN resource manager. This choice is primarily driven by the universality of the Spark engine for large-scale data sets, aligning with the core objective of integrating the system algorithm. Additionally, YARN facilitates dynamic scheduling of cluster resources, enhancing resource utilization.

**Figure 4:**
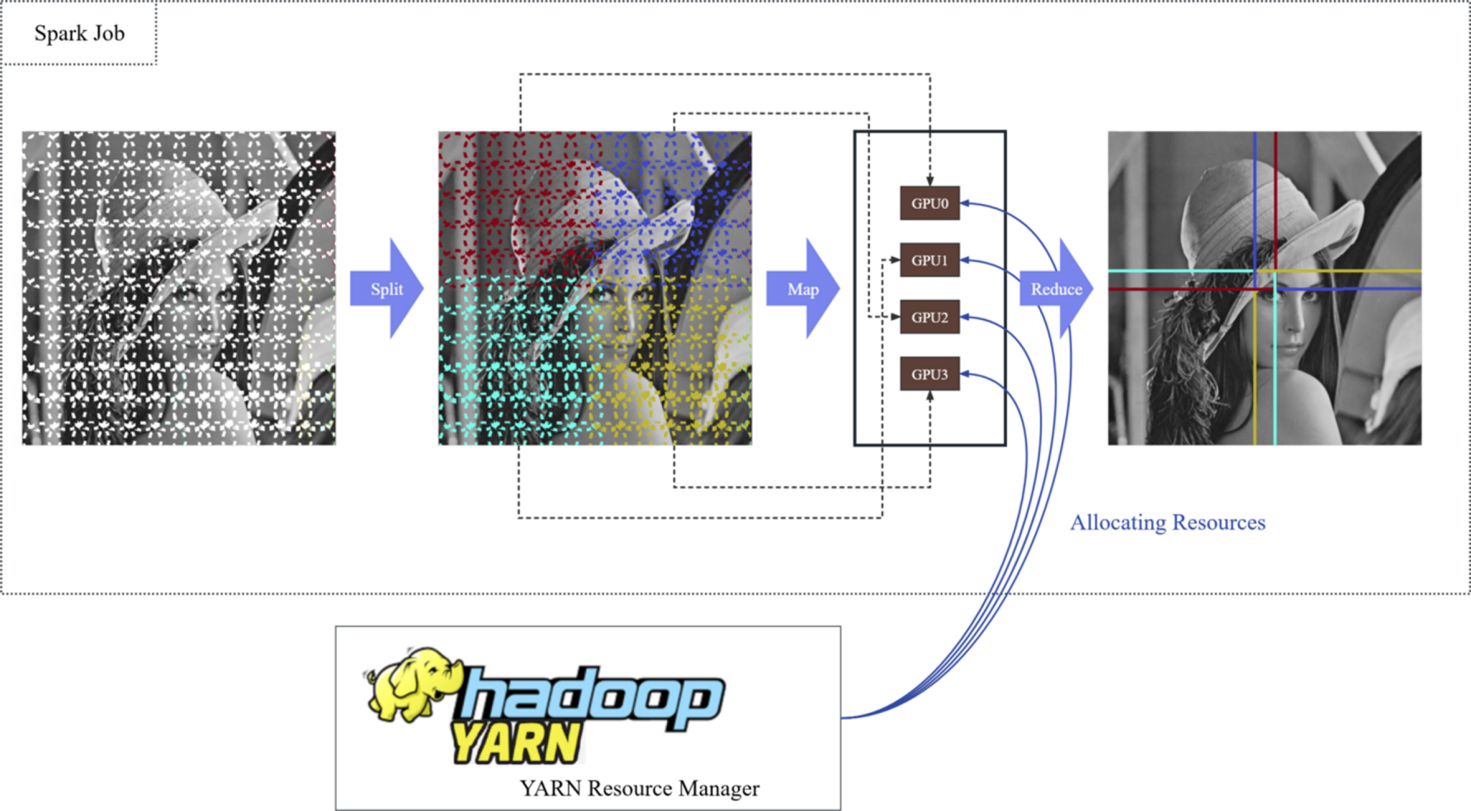
Illustration of parallel execution of ptychography imaging on a computing cluster with four GPUs.

As shown in Fig. 4 ▸, taking ptychography as an example, parallel optimization for ptychography involves five main steps (Favre-Nicolin *et al.*, 2020 ▸):

(1) Scan positions are split to different nodes using the *k*-means algorithm from the *scikit-learn* module (Pedregosa *et al.*, 2011 ▸).

(2) Each node loads a data set.

(3) Initialization of the object and probe in the driver, with each part mapped among executors.

(4) Independent execution of calculations among different executors.

(5) Reduce together different parts of the object.

The augmentation of system parallelism performance was addressed through the design of a critical middleware layer, comprising:

*Service coordination module (ZooKeeper)* (Hunt *et al.*, 2010 ▸): playing a crucial role in event listening and distributed locking within distributed systems, ensuring optimal system efficiency and collaboration.

*Big data computing engine (Spark)* (Zaharia *et al.*, 2010 ▸): selected to provide rapid, versatile and scalable capabilities for big data analytics. Implementation of imaging algorithms on Spark significantly enhances the parallelism and execution speed of the imaging process.

*Resource scheduling module (YARN)* (Vavilapalli *et al.*, 2013 ▸): furnishing the system with a well optimized computing resource scheduling mechanism, dynamically allocating resources based on application requirements for effective utilization.

*Application framework (Spring)*: serving as the foundational framework for the entire software system, providing robust and flexible support for application development and management.

The middleware layer efficiently supported various aspects of the system, including distributed processing, data management and application execution.

Through these detailed design and implementation strategies, we successfully realized user-friendliness and algorithm integration. The improvement of the parallelism efficiency is tested in the next section.

## 3. Results and discussion

Based on the methods proposed in the previous section, we conducted a series of tests to assess effectiveness. In this section, we outline the testing procedures, results and identified issues.

The performance and effectiveness of parallel systems or program parallelization are typically evaluated using various key indicators, including the following:

*Parallel acceleration ratio (PAR)* (Benzi & Damodaran, 2008 ▸): PAR represents the gain in speed achieved by parallel execution in comparison with serial execution. It is calculated as follows: 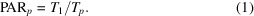
Here, *p* denotes the number of processors, *T*_1_ is the execution time of the sequential algorithm and *T*_*p*_ is the execution time of the parallel algorithm when *p* processors are utilized.

*Parallel efficiency (PEFF)* (Benzi & Damodaran, 2008 ▸): PEFF measures the efficiency of parallel execution and is given by the formula
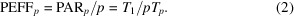
PEFF is typically expressed as the ratio of parallel execution time to serial execution time. A higher parallel efficiency value indicates more effective parallel execution.

These indicators provide valuable insights into the performance gains achieved through parallelization and help to gauge the efficiency of parallel computing systems.

We focused on typical algorithms commonly used in the ptychography imaging process. The choice of ptychography is particularly relevant given the growing maturity of the ptychography algorithm, its increasing applications and the escalating data volumes involved. This algorithm serves as a representative example of imaging algorithms that demand substantial computational resources, aligning with the challenges posed by data-intensive methods such as tomography and others mentioned earlier. The test was conducted on a computing cluster fitted with the following hardware configuration: two Intel Xeon Gold 6226R processors running at 2.90 GHz each, eight Nvidia A100-PCIE-40GB GPUs, 256 GB Samsung DDR4 memory modules. All nodes within the cluster are running the Ubuntu 20.04.5 LTS operating system.

Throughout the entire experimental process, we maintained a constant allocation of memory and CPU resources and utilized the same set of experimental data. Three ptychography algorithms were employed in various combinations: difference map (DM) algorithm, maximum-likelihood (ML) algorithm and alternating projection (AP) algorithm (DM*100 means the DM operator cycled 100 times). Within the same algorithmic combination, the only variable adjusted was the number of GPUs employed for parallel computation.

From Table 1 ▸, we are able to visualize the impact of the different number of GPUs in parallel on the experiment time, including data loading, preprocessing and algorithm execution, when the user performs experiments on SSRF’s hard X-ray nanoprobe beamline. It can be seen that GPU parallelism has a significant accelerating effect on both the data loading and preprocessing, and algorithm execution phases.

**Figure 5:**
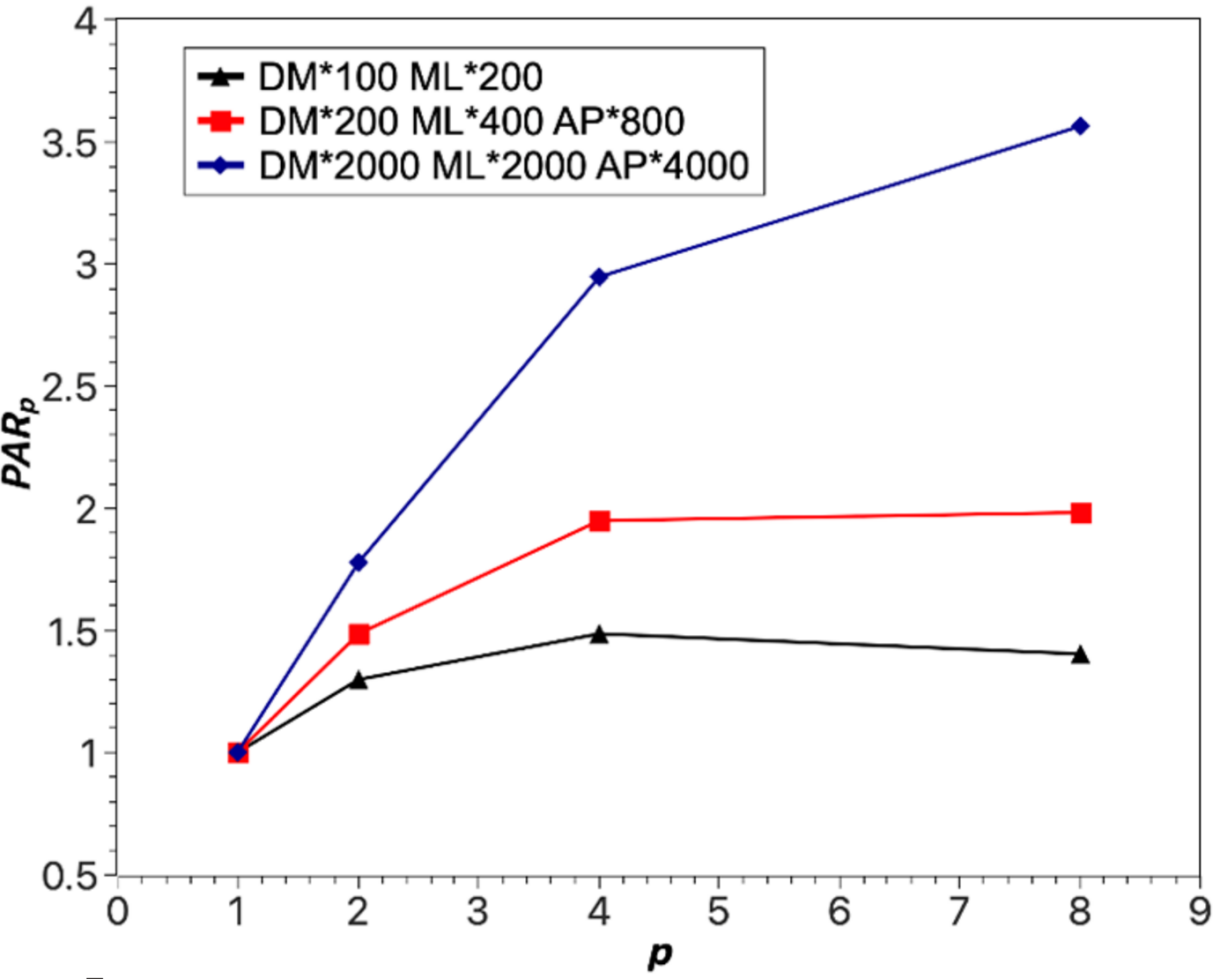
The influence of the number of processors (*p*) on the parallel acceleration ratio (PAR_*p*_).

As shown in Fig. 5 ▸, the parallel processing capability (PAR) of the software system progressively improves as the number of GPUs engaged in the computation increases. This enhancement is attributable to the increased capacity of multiple GPUs to simultaneously execute tasks, efficiently distributing the computational workload and augmenting the system’s processing speed. Remarkably, this improvement is particularly pronounced when the task’s computational load is relatively high. Therefore, increasing the number of GPUs is advantageous for enhancing the system’s parallel processing capability, resulting in reduced task completion times.

**Figure 6:**
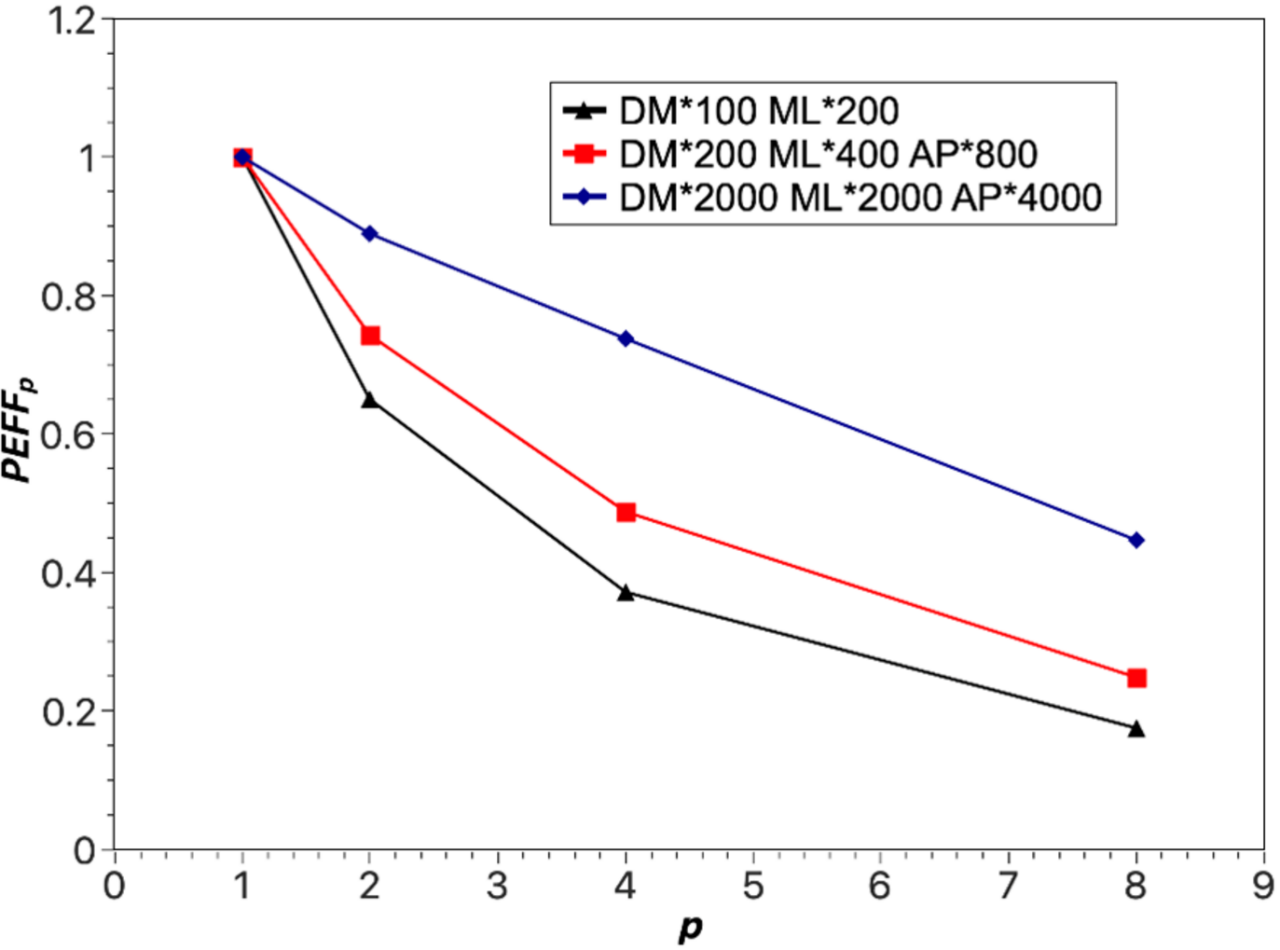
The influence of the number of processors (*p*) on the parallel efficiency (PEFF_*p*_).

However, as shown in Fig. 6 ▸, parallel efficiency gradually decreases as the number of GPUs involved in the computation increases. This decline can be attributed to the increased network communication overhead between parallel tasks, ultimately leading to a reduction in the overall system efficiency. However, in scenarios where the number of GPUs involved in the computation remains constant, increasing the computational load of the tasks can still yield improved parallel efficiency. With an increase in task computational load, the influence of network communication overhead on the overall task execution time decreases, resulting in an overall enhancement of the system’s efficiency with increasing task computational load.

In practical applications, achieving a balance between parallel processing efficiency and GPU usage is very important. This balance should be determined based on factors such as the task’s computational load and the available GPU resources. Specifically, when dealing with substantial task computational loads, increasing the number of GPUs can be a viable choice to enhance parallel processing capability. Conversely, for smaller task computational loads, it may be more appropriate to reduce the number of GPUs to improve parallel efficiency.

**Figure 7:**
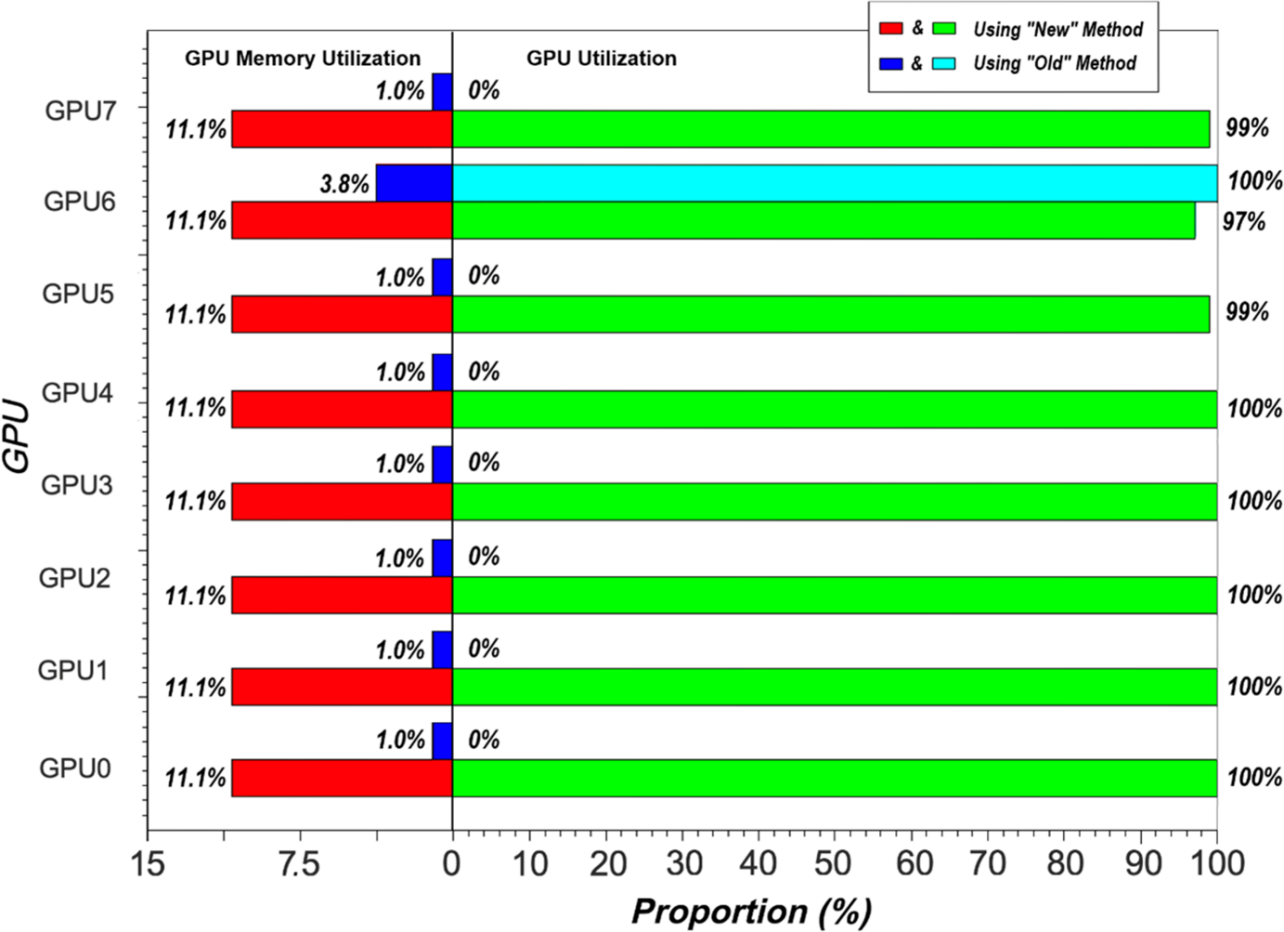
Comparison of parallel computing performance between the ‘New’ and ‘Old’ method.

In addition to the experiments on parallel computing efficiency mentioned above, we also evaluated the improvement in resource utilization achieved by our multi-GPU computing framework when executing the same algorithm under identical hardware configurations. As illustrated in Fig. 7 ▸, under the conditions of eight GPUs, the GPU utilization (UTL) increased from 100% to 795%, resulting in a 7.95-fold improvement. Similarly, the GPU memory utilization (MEM) increased from 3.8% to 88.8%, representing a 23.37-fold improvement.

Using ‘Old’ to denote the conventional phase retrieval imaging method that does not support multi-node clusters, and ‘New’ to represent the phase retrieval imaging method optimized and deployed on a multi-node cluster via our software system, Fig. 7 ▸ illustrates the performance metrics for resource utilization in a parallel computing environment with identical algorithm parameters. A comparison was conducted between the ‘Old’ and ‘New’ software methods using eight GPUs (GPU0 to GPU7). Only GPU6 is involved in the computation in the ‘Old’ method, so the GPU memory utilization (MEM) of the other seven GPUs is only about 1.0%, indicating that the GPU’s compute unit is not active, but the GPU memory may still be used to store small amounts of data or to perform non-compute intensive operations. In contrast, the ‘New’ method achieved 11.1% GPU memory utilization (MEM) on GPU0 and GPU7, indicating improved GPU memory utilization. The ‘Old’ method underutilized GPUs, with 0% GPU utilization (UTL), while the ‘New’ method consistently achieved 100% GPU utilization (UTL) on most GPUs, indicating more effective GPU resource usage. The results of this experiment demonstrate that our ‘New’ method exhibits a significant advantage in terms of GPU memory and computational resource utilization in a parallel computing environment. This finding holds particular significance for data-intensive computational imaging algorithms that require high computational performance.

Indeed, there are several valuable directions for further enhancing our system. Firstly, it is essential to allocate tasks in a balanced manner to ensure that each GPU task has a reasonable computational load. Secondly, it is crucial to optimize network communication to reduce communication overhead between parallel tasks. Lastly, implementing a performance scheduling strategy to dynamically allocate tasks based on GPU performance disparities can enhance overall system parallel efficiency. These aspects represent the ongoing focus of optimization and iteration for our system.

## 4. Summary

To address the growing data volumes and user requirements at the nanoprobe beamline, we have designed and implemented a software architecture. This architecture offers a streamlined, feature-rich and user-friendly interface to enhance the efficiency of algorithm and user experience. It leverages its thoughtfully crafted application layer, middleware and data layer to provide essential support for the efficient operation of the system. Moreover, the system integrates parallel computing optimizations, which significantly improve execution efficiency and resource utilization, especially in scenarios with complex imaging algorithms. It serves as an efficient, stable and reliable platform for large-scale synchrotron radiation data imaging.

In the future, there are several valuable directions to further enhance our system. Firstly, it is necessary to ensure balanced task allocation so that each GPU can handle a reasonable computational load. Secondly, the optimization of network communication to minimize communication overhead between parallel tasks is crucial. Lastly, the implementation of performance scheduling strategies that dynamically allocate tasks based on GPU performance differences can improve the overall parallel efficiency of the system. These aspects represent the focal points for the continuous optimization and iteration of our system.

## Figures and Tables

**Table 1:** The influence of the number of GPUs on the experiment time (loading, preprocessing, algorithm execution)

Algorithm	GPUs	Load and preprocess time (s)	Algorithm time (s)	All time (s)
DM*100 ML*200	2	976.87	325.43	1302.3
	4	627.34	171.24	798.58
	6	472.56	120.09	592.65
	8	392.51	100.24	492.75

DM*200 ML*400 AP*800	2	2349.07	984.23	3333.30
	4	1271.13	503.72	1774.85
	6	916.85	347.42	1264.27
	8	722.57	267.34	989.91
